# Supplementary material for: Effects of Complete Oral Motor Intervention and Nonnutritive Sucking Alone on the Feeding Performance of Premature Infants: A Systematic Review and Meta-Analysis
Source: Children (Basel). 2023 Dec 20;11(1):4. doi: 10.3390/children11010004 (PMC10814782; doi:10.3390/children11010004)
Supplement: Supplementary file 1 [file children-11-00004-s001.zip › children-2712565-supplementary.pdf]

Search Strategy

Database	#	Search syntax	Citations found
**1) Embase**	1	(Premature OR Preterm OR “Low birth weight” OR VLBW OR LBW OR Newborn OR Neonat* OR Infan*):ti,ab,kw,de	1914239
	2	“Prematurity”/exp OR “Low birth weight”/exp	186258
	3	(Oral OR “Oral motor” OR Oromotor OR Orocutaneous OR Prefeeding NEAR/3 Intervent* OR Stimulat* OR Train* OR Support*):ti,ab,kw,de	6488529
	4	(Non-nutritive OR “Non nutritive” OR nonnutritive OR Pacifier OR Dummy):ti,ab,kw,de	11705
	5	“Non nutritive sucking”/exp OR “Pacifier”/exp	1091
	6	(#1 OR #2) AND #3 AND (#4 OR #5) AND [embase]/lim	All: 405
	7	#6 AND ("randomized controlled trial"/de or "controlled clinical study"/de or "randomization"/de or "intermethod comparison"/de or "double blind procedure"/de or "human experiment"/de OR (random* or placebo or "parallel group$" or crossover or "cross over" or assigned or allocated or volunteer or volunteers):ti,ab OR (open NEAR/1 label):ti,ab OR ((double or single or doubly or singly) NEAR/1 (blind or blinded or blindly)):ti,ab OR ((assign* or match or matched or allocation) NEAR/5 (alternate or group$ or intervention$ or patient$ or subject$ or participant$)):ti,ab OR (controlled NEAR/7 (study or design or trial)):ti,ab OR (compare or compared or comparison or trial):ti OR ((evaluated or evaluate or evaluating or assessed or assess) and (compare or compared or comparing or comparison)):ab) NOT (((random* NEAR/1 sampl* NEAR/7 ("cross section*" or questionnaire$ or survey* or database$)):ti,ab not ("comparative study"/de or "controlled study"/de or "randomi$ed controlled":ti,ab or "randomly assigned":ti,ab)) OR ("cross-sectional study"/de not ("randomized controlled trial"/de or "controlled clinical study"/de or "controlled study"/de or "randomi$ed controlled":ti,ab or "control group$":ti,ab)) OR ((((case NEAR/1 control*) and random*) not "randomi$ed controlled"):ti,ab) OR (("systematic review" not (trial or study)):ti) OR ((nonrandom* not random*):ti,ab) OR ("random field*":ti,ab) OR (("random cluster" NEAR/3 sampl*):ti,ab) OR ((review:ab and review/it) not trial:ti) OR ("we searched":ab and (review:ti or review/it)) OR ("update review":ab) OR ((databases NEAR/4 searched):ab) OR ((rat or rats or mouse or mice or swine or porcine or murine or sheep or lambs or pigs or piglets or rabbit or rabbits or cat or cats or dog or dogs or cattle or bovine or monkey or monkeys or trout or marmoset*):ti and "animal experiment"/de) OR ("animal experiment"/de not ("human experiment"/de or "human"/de)))	**RCT:176**
**2) MEDLINE (PubMed)**	1	Premature OR Preterm OR “Low birth weight” OR VLBW OR LBW OR Newborn OR Neonat* OR Infan*	1837529
	2	“Infant, Premature”[mh] OR “Infant, Low Birth Weight”[mh]	92143
	3	((Oral OR “Oral motor” OR Oromotor OR Orocutaneous OR Prefeeding) AND (Intervent* OR Stimulat* OR Train* OR Support*))	475998
	4	Non-nutritive OR “Non nutritive” OR Nonnutritive OR Pacifier OR Dummy	7936
	5	“Placebos”[mh]	39493
	6	(#1 OR #2) AND #3 AND (#4 OR #5)	ALL: 411
	7	#6 AND (randomized controlled trial[pt] OR controlled clinical trial[pt] OR randomized[tiab] OR randomised[tiab] OR placebo[tiab] OR drug therapy[sh] OR randomly[tiab] OR trial[tiab] OR groups[tiab] NOT (animals [mh] NOT humans [mh]))	**RCT: 293**
